# Prevalence, spermatozoa, hormonal, and genetic evaluation of rare mosaic klinefelter syndrome patients in southern China

**DOI:** 10.3389/fgene.2025.1573292

**Published:** 2025-06-10

**Authors:** Derong Li, Yuqing Lai, Yujuan Liao, Jinjie Pan, Yudi Luo, Yunning Liang, Bowen Luo, Lingling Zhu, Guosheng Deng, Xiang Li, Keng Feng, Feifei Lei, Liuping Lan

**Affiliations:** ^1^ Reproductive Medicine Center, Yulin Maternal and Child Healthcare Hospital, Yulin, Guangxi, China; ^2^ Obetrics Outpatient Clinic Yulin Maternal and Child Healthcare Hospital, Yulin, Guangxi, China; ^3^ Clinical Laboratory, Yulin Maternal and Child Healthcare Hospital, Yulin, Guangxi, China

**Keywords:** Klinefelter syndrome (KS), chromosomes, male infertility, reproductive hormones, azoospermia

## Abstract

**Background:**

Klinefelter syndrome (KS) is the most common genetic cause of male infertility in humans. Mosaic KS is a subtype of KS. Due to its low prevalence, the lack of typical clinical signs, and the limited professional awareness of the syndrome, many cases of mosaic KS remain undiagnosed.

**Objective:**

To investigate the prevalence of rare mosaic KS, karyotype characteristics, reproductive hormone levels, and sperm quality in southern China, and to enrich our knowledge of patients with mosaic KS.

**Methods:**

The chromosome results of 8,110 cases of infertile male patients attending the Reproductive Center of Yulin Maternal and Child Health Hospital from January 2018 to July 2024 were retrospectively analyzed. Semen, sex hormone tests, and Y chromosome microdeletions were analyzed in fourteen selected patients with a diagnosis of mosaic KS.

**Results:**

Among the 8,110 male infertility patients, a total of 0.703% (57/8,110) were diagnosed with KS. Of these, 0.530% (43/8,110) had the typical non-mosaic chromosomal karyotype of 47, XXY, while 0.172% (14/8,110) demonstrated mosaicism. Non-mosaic KS accounted for 75.44% (43/57), whereas mosaic KS accounted for 24.56% (14/57). Among the fourteen patients diagnosed with mosaic KS, the predominant chromosomal karyotype was 47,XXY/46,XY, observed in eleven patients, accounting for 78.57% of the cases. Additionally, one patient exhibited a chromosomal karyotype of 47,XXY [29]/46,XX [78]. Another patient had 47,XXY [92]/48,XXXY [3]/46,XY [5], and a third patient presented with 47,XXY [87]/46,XX [3]/46,XY [2]. The semen analysis of individuals with mosaic KS revealed two cases of azoospermia, one case of cryptozoospermia, one case of severe oligospermia, one case of oligospermia, and ten cases of normal sperm. The results of the sex hormone analysis revealed abnormal increases in serum follicle-stimulating hormone (FSH) levels in four patients diagnosed with mosaic KS. Additionally, two patients with mosaic KS exhibited higher luteinizing hormone (LH) and testosterone (TT) levels compared to the normal range, while four patients had lower estradiol (E2) levels than the normal range. Among the fourteen patients with mosaic KS, four couples underwent assisted reproductive technology at our hospital for fertility assistance. Of these four couples, two successfully gave birth to a healthy child.

**Conclusion:**

The prevalence of mosaic KS among male infertility patients in southern China is 0.172% (14/8,110), with the predominant chromosomal karyotype being 47,XXY/46,XY, accounting for 78.57% (11/14) of the cases. Patients with mosaic KS may present with various sperm conditions, including azoospermia, cryptozoospermia, severe oligozoospermia, oligozoospermia, and normal sperm. There is a notable correlation between sperm count and the number of abnormal cell karyotypes in these patients; specifically, a higher number of abnormal chromosomal mosaic cells is associated with a lower sperm count. In comparison to non-mosaic KS patients, those with mosaic KS exhibit a lower rate of azoospermia. In summary, patients with rare mosaic KS in southern China demonstrate significant heterogeneity in sperm production, hormonal levels, and genetics. These patients can achieve biological fatherhood through assisted reproductive techniques, and mosaic KS does not appear to impact the success rate of these techniques. However, due to the low prevalence and limited sample size, more data is necessary to confirm these findings.

## 1 Introduction

KS is a disease characterized by the presence of an extra X chromosome in males. It is the most common sex chromosome abnormality in males, typically presenting with a chromosomal karyotype of 47XXY. The incidence of KS in newborns ranges from 0.04% to 0.23%, with a prevalence of 11% among cases of azoospermia (Lanfranco et al., 2004; Bojesen et al., 2003; Herlihy et al., 2011). KS is classified into three types: mosaic KS, non-mosaic KS, and variant KS. Approximately 90% of individuals with KS have a chromosomal karyotype of non-mosaic 47,XXY, while less than 10% exhibit mosaic or variant forms of the syndrome (Lanfranco et al., 2004; Bearelly and Oates, 2019). The typical clinical manifestations of patients with non-mosaic KS(47,XXY) include small testicles, infertility, hypogonadism, and cognitive impairment, among other symptoms (Samango-Sprouse et al., 2019; Aksglaede et al., 2013; Akcan et al., 2018). Structural abnormalities, such as 47,XX,der(Y) and 47,XY,der(X), as well as chromosomal aneuploidies with two or more extra chromosomes (48, XXXY and 49, XXXXY), are often considered variants KS because of these shared features. However, the increased risks of congenital malformations, additional medical complications, and more complex psychological issues associated with 48,XXYY, 48,XXXY, and 49,XXXXY make it essential to distinguish these conditions from 47,XXY for the benefit of affected patients. Mosaic KS refers to the presence of cells with two or more different karyotypes within the same individual, such as 46XY/47XXY or 46XY/45X/47XXY.The clinical phenotype of patients with mosaic KS is characterized by mostly mild and rare symptoms, resulting in a lack of specificity. This lack of specificity often leads to a low diagnosis rate and frequently causes delays in both diagnosis and treatment (Aksglaede et al., 2020). Considering the current rarity of mosaic KS and the limited availability of related literature, this study retrospectively analyzed 14 patients with mosaic KS in the southern region of China. The analysis focused on the patients’ chromosomal karyotypes, serum sex hormone levels, and sperm quality. The aim of this study is to provide a foundation for clinical diagnosis, genetic counseling, and treatment options for KS.

## 2 Methods

A retrospective analysis was conducted on the chromosomes of 8,110 male infertility patients who visited the Reproductive Center of Yulin Maternal and Child Health Hospital from January 2018 to June 2024. Retrospective analyses were performed on sex hormones, chromosomal karyotypes, Y chromosome microdeletions, and semen samples from patients diagnosed with KS based on their chromosomal karyotypes. Serum levels of FSH(range:0.95–11.95 mIU/mL), LH (range:0.57–12.07 mIU/mL), PRL (range:1.42–9.23 ng/mL), TT (range:1.42–9.23 ng/mL), and E2 (range:11–44 pg/mL) were measured using chemiluminescent immunoassay and radioimmunoassay techniques in the Laboratory Department of Yulin Maternal and Child Health Hospital. Semen analysis was conducted at the Reproductive Laboratory of Yulin Maternal and Child Health Hospital in accordance with the standards established by the World Health Organization (WHO). Sperm count, sperm concentration, and motility were assessed using computer-aided semen analysis. All andrology laboratories employ validated methods and implement their own quality control procedures. Y chromosome microdeletions were analyzed for detection using PCR amplification in the PCR Laboratory of Yulin Maternal and Child Health Hospital. The guidelines provided by the European Academy of Andrology and European Molecular Genetics were followed for the initial screening of AZF deficiency. Six loci were selected for the identification of Y microdeletions: SY84/SY86 (AZFa), SY127/SY134 (AZFb), and SY254/SY255 (AZFc). Chromosomal karyotype analysis was conducted at the Cytogenetic Laboratory of Yulin Maternal and Child Health Hospital using the G banding technique to identify karyotypes. At a resolution of 320–400 bands per haploid chromosome set in each group, a minimum of 20 metaphases were analyzed for each patient, with up to 100 metaphases analyzed in the case of chimeras. All chromosomal karyotype analyses were performed in the same laboratory according to standardized protocols. The chromosomal karyotypes were described following the guidelines of the International System for Human Cytogenomic Nomenclature (ISCN, 2020). This is a retrospective study conducted in accordance with the Helsinki principles, ensuring that the privacy and interests of the patients were upheld. Consequently, the Ethics Committee of Yulin Maternal and Child Health Hospital approved this study and obtain the patient’s informed consent.

## 3 Result

**FIGURE 1:**
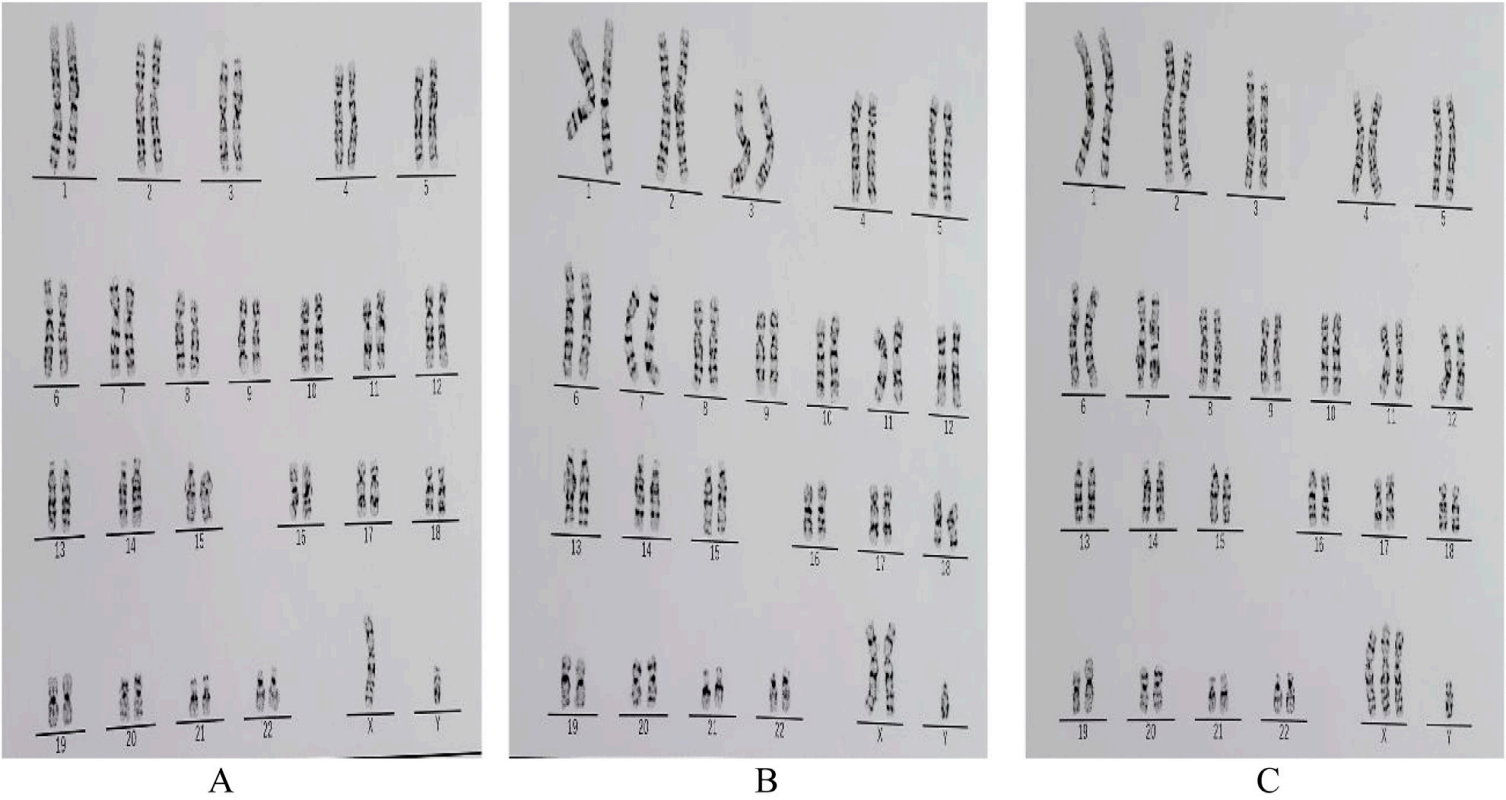
Karyograms of the patients with 47, XXY/48, XXXY/46, XY **(A)** 46, XY. **(B)** 47, XXY **(C)** 48, XXXY.

In the present study, the demographic characteristics and general features of the infertile patients are presented in Table 1. There were significant differences in the clinical characteristics between patients with mosaic KS and those with non-mosaic KS. Among the 8,110 male infertility patients, a total of 0.703% (57/8,110) were diagnosed with KS. Of these, 0.530% (43/8,110) had the typical non-mosaic chromosomal karyotype of 47, XXY. The diagnosis of non-mosaic KS (47,XXY) accounted for 75.44% (43/57) of KS cases, with an average age at diagnosis of 31.20 ± 5.07 years and an azoospermia rate of 95.65% (22/23). In contrast, the incidence rate of mosaic KS was 0.172% (14/8,110), representing 24.56% (14/57) of KS cases, with an average age at diagnosis of 36.35 ± 6.05 years and an azoospermia rate of 14.29% (2/14). The most common chromosomal karyotype observed in patients with KS is 47,XXY/46,XY, which accounts for 78.57% (11/14) of cases. In addition, there was one case with a karyotype of 47,XXY [29]/46,XX [78], one case with a karyotype of 47,XXY [92]/48,XXXY [3]/46,XY [5], and another case with a karyotype of 47,XXY [87]/46,XX [3]/46,XY [2]. All patients showed normal Y chromosome microdeletions (Table 2; Figure 1).

**TABLE 1:** Characteristics of the mosaic KS and non-mosaic KS patients.

Characteristics	Mosaic KS	non-mosaic KS	All KS
Number of patients	14	43	57
Diagnosis mean age(year)	36.35±6.05	31.20±5.07	32.44±6.12
Prevalence	0.172%(14/8110)	0.530%(43/8110)	0.703%(57/8110)
Klinefelter proportion of composition	24.56%(14/57)	75.44%(43/57)	100%(57/57)
Azoospermia	14.29%(2/14)	95.65%(22/23)	64.86%(24/37)
Normal percentage of Y chromosome microdeletions	100%(13/13)	100%(28/28)	100%(41/41)
Abnormality percentage of FSH(>11.95 mIU/ml)	28.57%(4/14)	97.14%(34/35)	77.55%(38/49)
Abnormality percentage of LH(>12.07 mIU/ml)	16.67%(2/12)	85.71%(30/35)	68.08%(32/47)
Abnormality percentage of T (<1.42 ng/mL)	16.67%(2/12)	31.42%(11/35)	27.65%(13/47)
Abnormality percentage of PRL(>19.4 ng/mL)	7.14%(1/14)	5.71%(2/35)	6.12%(3/49)

KS, Klinefelter syndrome; FSH, follicle stimulating hormone; LH, luteinizing hormone; TT, testosterone; PRL, prolactin; E2, estradiol.

**TABLE 2:** Characteristics of the mosaic KS patients.

	Karyotype	Semen analysis	Y chromosome microdeletions	FSH, mIU/mL	LH, mIU/mL	TT, ng/mL	PRL, ng/mL	E2, pg/mL
1	47,XXY[2]/46,XY[98]	normal	normal	5.03	4.53	3.79	13.04	NR
2	47,XXY[2]/46,XY[98]	normal	normal	2.15	1.42	3.46	7.12	< 10
3	47,XXY[2]/46,XY[98]	normal	normal	8.2	2.62	4.64	6.79	28
4	47,XXY[2]/46,XY[98]	Asthenospermia	normal	4.69	4.3	5.63	6.44	25
5	47,XXY[2]/46,XY[98]	Asthenospermia	normal	5.33	2.24	3.49	17.68	< 10
6	47,XXY[2]/46,XY[97]	normal	normal	2.34	1.23	6.03	4.57	10
7	47,XXY[2]/46,XY[99]	normal	normal	5.01	2.25	6.45	12.37	26
8	47,XXY[3]/46,XY[97]	oligoasthenozoospermia	normal	5.82	3.44	4.81	18.97	37
9	47,XXY[3]/46,XY[96]	normal	normal	3.1	4.33	16.86	NR	151
10	47,XXY[3]/46,XY[87]	oligozoospermia	normal	24.44	7.84	2.87	6.34	23
11	47,XXY[29]/46,XX[78]	cryptozoospermia	NR	32.49	16.34	5.65	29.03	38
12	47,XXY[87]/46,XX[3]/46,XY[2]	Azoospermia	normal	47.73	13.52	0.63	8.68	< 10
13	47,XXY[92]/48,XXXY[3]/46,XY[5]	Azoospermia	normal	25.68	8.02	0.44	11.79	< 10
14	47,XXY,t(9;10)(p22;q26.1)[2]/46,XY,t(9;10)(p22;q26.1)[98]	normal	normal	3.46	2.49	5.26	12.66	23

KS, Klinefelter syndrome; FSH, follicle stimulating hormone; LH, luteinizing hormone; TT, testosterone; PRL, prolactin; E2, estradiol NR, not reported.

The semen analysis results for patients diagnosed with mosaic KS showed the following findings: two cases of azoospermia, one case of cryptozoospermia, one case of severe oligozoospermia, one case of oligozoospermia, and ten cases of normozoospermia. Azoospermia was observed in mosaic Klinefelter syndrome (KS) patients with the following karyotypes: 47,XXY [92], 48,XXXY [3], 46,XY [5], and 47,XXY [87], 46,XX [3], 46,XY [2]. The incidence of azoospermia in patients with mosaic KS was 14.29% (2/14), which is significantly lower than the 95.65% (22/23) observed in patients with non-mosaic KS.

The results of the sex hormone analysis indicated that the serum FSH levels in four patients with mosaic KS were higher than the normal range. Additionally, the LH and TT levels in two patients with mosaic KS exceeded the normal values, while the E2 levels in four patients with mosaic KS were lower than the normal range. Compared to patients with non-mosaic KS, the abnormal rate of FSH and LH hormones in patients with mosaic KS was significantly lower.

Among the fourteen patients diagnosed with KS, eight sought assisted reproductive technology (ART) for fertility assistance at our hospital. Of these, four patients completed the ART process. The wives of two patients achieved successful pregnancies after a single embryo transfer, while the wife of another patient became pregnant after three embryo transfers. Among these couples, the wives of two patients delivered healthy babies; however, one patient’s wife experienced a miscarriage due to fetal anemia during the second trimester.

## 4 Discussion

KS is the most prevalent sex chromosome disorder affecting males. The numerical chromosome aberrations in Klinefelter’s syndrome result from non-disjunction, which can occur during meiotic divisions in germ cell development or during early embryonic mitotic cell divisions. The presence of an extra X chromosome can be inherited from either the father or the mother (Hall et al., 2006). KS is classified into three types: mosaic KS, non-mosaic KS, and variant KS. The typical clinical manifestations of patients with non-mosaic KS include small testicles, infertility, hypogonadism, and cognitive impairment, among other symptoms (Rocher et al., 2016). Some patients may exhibit abnormalities in their reproductive organs, including undescended testicles (cryptorchidism), hypospadias, and micropenis (Akcan et al., 2018). KS significantly affects male fertility and psychological wellbeing. It has been reported that the incidence of KS is influenced by race and geographic region to some extent. According to a recent study, the prevalence among newborns in Denmark is 0.028% (Bojesen et al., 2003). Whereas in Australia, it is 0.23% (Herlihy et al., 2011). Additionally, the prevalence among azoospermic patients ranges from 10% to 12%, and there is a trend of increasing incidence each year (Forti et al., 2010). Research on KS mainly focuses on non-mosaic KS patients. Currently, there is a lack of data regarding mosaic KS and a large-scale Chinese population. China is the most populous country in the world, with a population of 1.4 billion and 56 recognized ethnic groups. The only available report in China addresses the prevalence of KS among individuals with severe oligoasthenozoospermia in eastern China, which accounts for 1,072 out of 10,286 cases (10.35%) (Chen et al., 2017). No reports on the prevalence of KS in southern China have been identified.

This study is the first to report on the characteristics of mosaic KS in southern China. The study included 8,110 adult male infertile patients from southern China. The prevalence of KS was found to be 0.703% (57/8110), which is significantly higher than the rates observed in countries such as Denmark and Australia. The percentage of patients with non-mosaic KS among individuals with KS in southern China was 75.44% (43/57), significantly lower than the 80%–90% observed in other ethnic groups and regions (Bearelly and Oates, 2019). Additionally, the prevalence of mosaic KS in southern China was 0.172% (14/8110), and the proportion of mosaic KS among KS patients was 24.56% (14/57). This rate is higher than the 10%–20% observed in other ethnic groups and regions (Bearelly and Oates, 2019). The main chromosomal karyotype observed in patients with mosaic KS was 47,XXY/46,XY, accounting for 78.57% (11/14) of the cases. The findings of this study were inconsistent with those of other research, which may be attributed to variations in geographic regions and ethnic groups. The southern and eastern regions of China are separated by approximately 2,000 km, resulting in significant differences in geography, population, genetic backgrounds, as well as dietary and lifestyle habits. These factors may influence the results. Furthermore, the data for this study were obtained from a male infertility clinic, whereas other studies may have drawn from a broader range of data sources beyond male infertility clinics. Mosaic KS refers to a chromosomal karyotype in which patients have cells with different chromosomal compositions. In addition to the typical 47,XXY karyotype, these patients may also possess cells with other karyotypes, such as 46,XY and 47,XXXY, among others. The most common chromosomal karyotype associated with mosaic KS is 46,XY/47,XXY. The data from our current study align with this finding. The data obtained from this study indicate that the predominant karyotype observed in patients with mosaic KS in southern China is 46,XY/47,XXY, which accounts for 78.57% (11/14). In addition, there was one case each of patients with the chromosomal karyotypes 47,XXY [29]/46,XX [78], 47,XY [92]/48,XXXY [3]/46,XY [5], and 47,XXY [87]/46,XX [3]/46,XY [2]. These patients are classified as having rare types of KS. Currently, the number of reported cases, both domestically and internationally, is relatively small, and the underlying cause remains unclear. However, some researchers speculate that this may be attributed to the presence of the Y chromosome in the majority of gonadal cells in these patients, which results in a male clinical phenotype (Shehab et al., 2018). This study reports that the prevalence of mosaic KS was 0.172% (14/8110), significantly lower than the prevalence of non-mosaic KS at 0.530% (43/8110). Meanwhile, the average age of patients with mosaic KS was 36.35 ± 6.05 years (range: 28–48 years). The mean age of KS patients was 31.20 ± 5.07 years (range: 18–43 years), and it was found to be significantly lower than the mean age of mosaic KS patients. Compared to patients with non-mosaic KS, the prevalence of mosaic KS is lower, and the age of diagnosis is higher. This discrepancy may be attributed to the milder clinical phenotypes and the lack of clinical specificity in patients with mosaic KS, which often results in a lower rate of clinical diagnosis. Reports indicate that approximately two-thirds of patients are misdiagnosed, leading to delays in treatment (Bird and Hurren, 2016). In this study, the majority of patients with KS were diagnosed while being treated for male infertility, with an average age of diagnosis of 32.44 ± 6.12 years. Therefore, it is crucial to enhance our understanding of KS. Most patients with non-mosaic KS typically exhibit symptoms such as azoospermia, small testicles, and infertility. It is important to note that the occurrence of azoospermia in patients with KS is not associated with Y chromosome microdeletions (Sciarra et al., 2019), as our current study has also substantiated. In our study, the azoospermia rate among patients with non-mosaic KS reached 95.65% (22/23); and the Y chromosome microdeletions were found to be completely normal. However, patients with mosaic KS are rare and display a diverse array of clinical manifestations. There is a limited number of reports available on this topic. In our study, we report the characteristics of 14 patients with KS, enhancing our understanding of this condition. Among the 11 patients with a chromosomal karyotype of 46, XX/47, XXY, all exhibited sperm production. Additionally, these patients demonstrated normal levels of six sex hormones and showed no observable deletions of the Y chromosome, consistent with individuals who do not have KS. In these 11 cases of mosaic KS, the number of 47,XXY cells in the mosaic did not exceed 10. The completely normal clinical phenotype observed in these cases may be associated with the lower number of 47,XXY cells present in the mosaic. Research has shown that the clinical manifestations and sperm count in mosaic KS correlate with the number of mosaic cells. Generally, a higher number of mosaic cells corresponds to a more significant impact on the clinical presentation of the patients (Koga et al., 2007). The other three patients in our current study also supported this point from a different perspective. The chromosomal karyotypes of the other three patients with mosaic KS were as follows: 47,XY [92]/48,XXXY [3]/46,XY [5],47,XXY [87]/46,XX [3]/46,XY [2], and 47,XXY [29]/46,XX [78]. Notably, all of them exhibited azoospermia. Among these three patients with mosaic KS, the normal chromosomal karyotype of 46,XY was significantly reduced, with fewer than 10 cells observed. Additionally, rare karyotypes, such as 48,XXXY and 46,XX, were identified. Furthermore, these patients displayed abnormal levels of sex hormones, primarily characterized by elevated FSH and LH values.

Endocrine changes are one of the characteristics of non-mosaic KS, which is manifested by significantly elevated levels of FSH and luteinizing hormone, along with decreased or normal levels of TT. The testis is a vital organ responsible for the production of sperm and the synthesis of sex hormones. It is primarily composed of spermatogenic cells, Sertoli cells, and Leydig cells, each performing specific functions under the strict regulation of the hypothalamic-pituitary-testicular axis. Any alterations in the cellular components of the testis can affect its size and the levels of reproductive hormones, potentially leading to an imbalance in the regulation of the hypothalamus and pituitary gland. The production of sperm requires the presence of gonadotropins, specifically FSH and LH, as well as TT. FSH directly stimulates spermatogenesis by acting on Sertoli cells, while LH indirectly influences this process by first promoting the production of TT in Leydig cells. TT then acts on Sertoli cells in the seminiferous tubules and peritubular cells to facilitate spermatogenesis. Additionally, FSH is crucial for stimulating DNA synthesis in spermatogonia (Anderson et al., 1997). Therefore, elevated serum FSH levels in infertile men are considered a reliable indicator of impaired spermatogenic function and are often associated with azoospermia and severe oligospermia (Jarvi et al., 2010). In this study, among the fourteen cases of patients with mosaic KS, the rate of abnormal elevation in FSH was 28.57% (4/14), and the rate of abnormal elevation in LH was 16.67% (2/12). These rates were significantly lower than the rates of 97.14% (34/35) and 85.71% (30/35) observed in patients with non-mosaic KS. The presence of an additional X chromosome may lead to various complications. This extra chromosome interferes with the normal function of the Y chromosome, resulting in the degeneration and fibrosis of the seminiferous tubules. Consequently, this condition reduces the secretion of certain factors that normally inhibit the release of pituitary FSH and LH, leading to significantly elevated concentrations of FSH and LH in the serum. As a result, the development of the male reproductive system is adversely affected. Therefore, measuring sex hormone levels can be considered one of the criteria for the initial diagnosis of patients with KS. Additionally, these measurements provide a crucial foundation for administering hormone treatment to patients with KS in a clinical setting.

Among the fourteen patients diagnosed with mosaic KS, eight underwent assisted reproductive technology (ART) to assist with pregnancy. Four of these patients completed the ART process. The wives of two patients achieved successful pregnancies after a single embryo transfer, while the wife of another patient became pregnant after three embryo transfers. Among these couples, the wives of two patients delivered healthy babies; however, one patient’s wife experienced a miscarriage due to fetal anemia during the second trimester. For patients with mosaic KS, their cellular chromosomal karyotypes do not appear to affect the success rate of *in vitro* fertilization.

This research is subject to several limitations. Primarily, it is a retrospective study, and essential patient metrics, including height, weight, and testicular size, were not recorded. These variables are crucial for a comprehensive understanding of KS. Additionally, the scarcity of individuals diagnosed with mosaic KS results in a limited dataset, necessitating further validation of the findings through larger population studies, multi-center research, and the inclusion of diverse ethnic groups to enhance the generalizability of the conclusions.

## 5 Conclusion

The prevalence of KS among male infertility patients in South China is 0.703% (57/8,110), while the prevalence of mosaic KS is 0.172% (14/8,110). In South China, the proportion of homozygous KS patients is 75.44% (43/57), which is significantly lower than the 80%–90% observed in other ethnic groups. The main karyotype found in patients with mosaic KS in South China is 46, XY/47, XXY, accounting for 78.57% (11/14). The azoospermia rate among patients with mosaic KS in South China is 14.29% (2/14), which is significantly lower than the 95.65% (22/23) observed in patients with non-mosaic KS. Patients with mosaic KS may present with azoospermia, cryptozoospermia, severe oligospermia, oligospermia, or normal sperm counts. The clinical phenotype of patients with mosaic KS may be associated with the karyotypic mosaicism of the cell nucleus. A greater degree of karyotypic mosaicism is correlated with a higher likelihood of azoospermia. Compared to patients with non-mosaic KS, those with mosaic KS exhibit a lower incidence of azoospermia.

In conclusion, there is significant heterogeneity in spermatogenesis, hormones, and genetic assessment among rare mosaic KS patients in southern China. The majority of these patients are able to achieve biological fatherhood through the use of modern assisted reproductive technology. It has been observed that mosaic KS patients do not have an impact on the success rate of assisted reproductive technology. However, due to the low prevalence and small sample size of these patients, further data is required to provide conclusive evidence.

## Data Availability

The original contributions presented in the study are included in the article/supplementary material, further inquiries can be directed to the corresponding authors.

## Abbreviations

KS, Klinefelter syndrome; FSH, follicle-stimulating hormone; LH, luteinizing hormone; TT, testosterone; PRL, prolactin; E2, estradiol.
